# Supplementary material for: Mechanism and inhibition of Streptococcus pneumoniae IgA1 protease
Source: Nat Commun. 2020 Nov 27;11:6063. doi: 10.1038/s41467-020-19887-3 (PMC7695701; doi:10.1038/s41467-020-19887-3)
Supplement: Supplementary file 1 — Supplementary Information [file 41467_2020_19887_MOESM1_ESM.pdf]

Supplementary Information

Mechanism and inhibition of *Streptococcus pneumoniae* IgA1 protease.

Wang et al

Supplementary Figures.

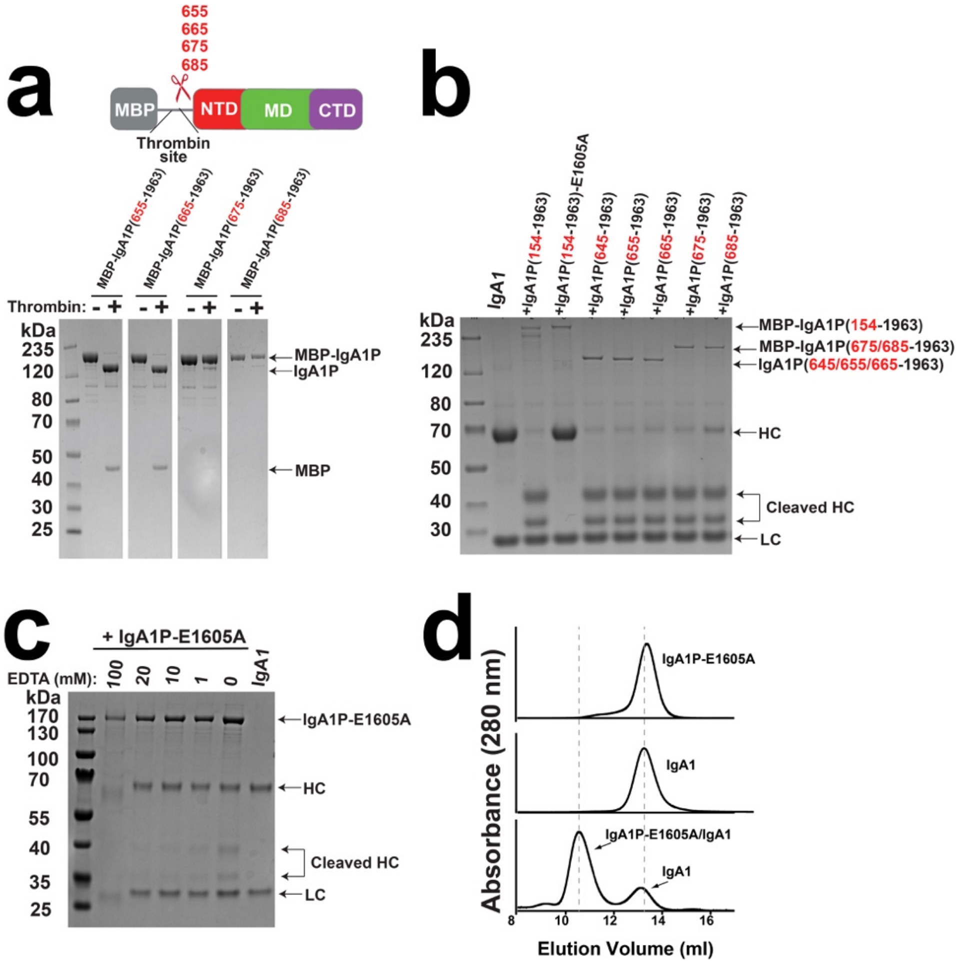

Supplementary Figure 1

Supplementary Fig. 1. Identifying the catalytic region of *S. pneumoniae* IgA1P and optimizing conditions for trapping the IgA1P/IgA1 substrate complex. a) Thrombin is unable to excise the N-terminally tagged MBP-IgA1P recombinant fusion proteins unless the N-terminus begins prior to residue 665. Multiple recombinant proteins were engineered with varying start positions (red) based on our previously collected mass spectrometry data and predicted secondary structure [1]. All constructs were designed with an N-terminal MBP tag, a thrombin site to remove MBP, and a C-terminal 6xHis tag. The structure is shown as a cartoon with the four

IgA1P start sites in red (top) and the SDS-PAGE analysis of proteins in the absence and presence of thrombin (bottom). All gels were collected and processed simultaneously. b) Catalytic activities of engineered IgA1P constructs. Conditions were as previously described [1]. c) Stoichiometric amounts of IgA1P-E1605A (residues 665-1963) and IgA1 substrate (30 μM final concentration) were incubated for 1 hour at 37°C under varying concentrations of EDTA. Results indicate that the E1605A construct is still partially active, but its activity is diminished in the presence of EDTA. d) Analytical size exclusion chromatography using a 23.5 ml analytical Superdex 200 (GE Healthcare). Chromatogram traces of the IgA1P-E1605A alone (top), the purified IgA1 monomer (middle), and the IgA1P-E1605A/IgA1 complex with the addition of 1 mM EDTA in the running buffer (bottom) are shown. All SDS-PAGE gels are representative of two independent experiments.

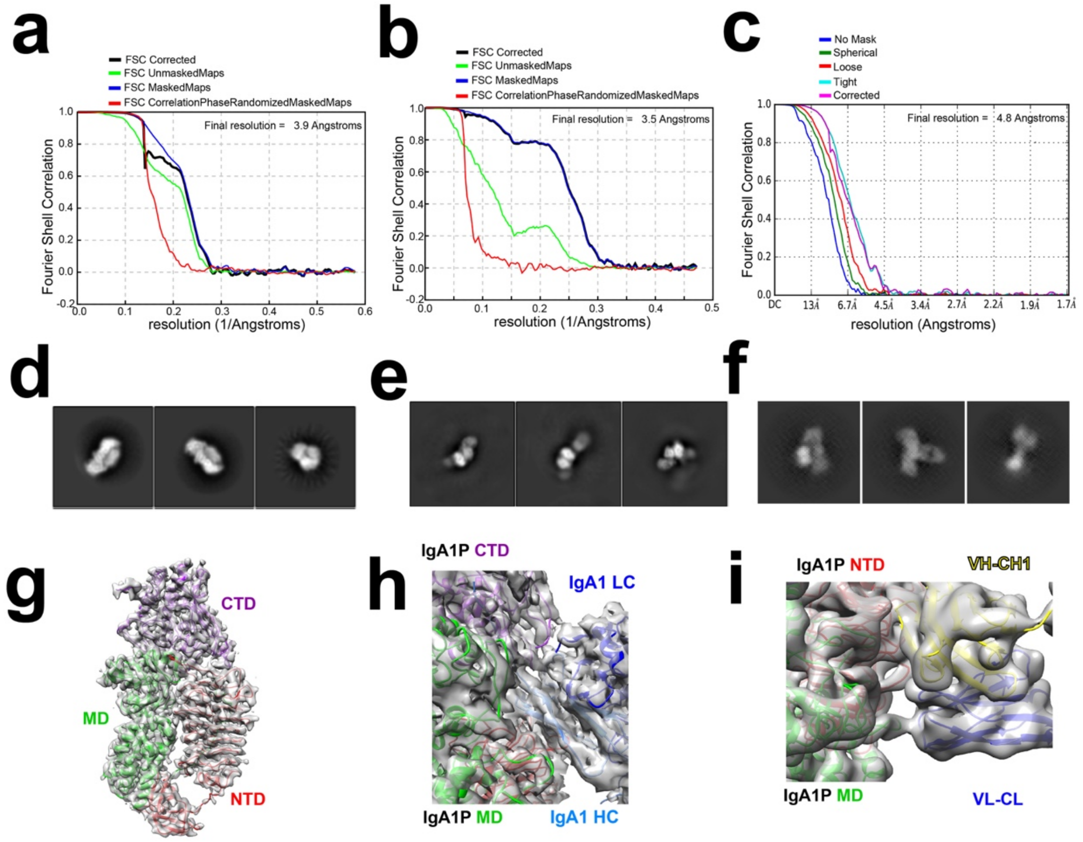

Supplementary Figure 2

Supplementary Fig. 2. **Resolution of the cryo-EM data for the *S. pneumoniae* IgA1P and IgA1P-E1605A/IgA1 complex**. a) FSC curves for IgA1P (residues 665-1963) exported from RELION. b) FSC curves for the IgA1P-E1605A/IgA1 complex exported from RELION. c) FSC curves for the IgA1P/mAb complex exported from cryoSPARC. d) Representative 2D class averages of IgA1P residues 665-1963. e) Representative 2D class averages of the IgA1P/IgA1 complex. f) Representative 2D class averages of the IgA1P/mAb complex. g) Density and corresponding model for the entirety of IgA1P. h) Density and corresponding model of the interface between IgA1P/IgA1 surround the IgA1 substrate hinge region. i) Density and corresponding model of the IgA1P/mAb interface.

a

```
               10        20        30         40        50
           123 4567890123456789012345678901  2345678901234567890123456
           a.a .aa..aaaaa..a..........a.aaa  aaaa...aaa..a....aa.aaa.aa
3m8o_HC:   EVQ-LVESGGGLVQPGGSLKLSCAASGFTLSG--SNVHWVRQASGKGLEWVGRIKRNAES 57
1iga_HC:   QVKLLEQSGAEVKKPGASVKVSCKASGYSFTS--YGLHWVRQAPGQRLEWMGWISAG--T 56
Sequ 1:    --------------------------GFTFST--YWMTWVRQAPGKGLEWVATINQD--G
Sequ 2:    --------------------------GFTFST--YWMTWVRQAPGKGLEWVATINQD--G
Sequ 3:    --------------------------GGFVSSGNYYWTWIRQPPGKGLEWIGSIYYS--G
Sequ 4:    --------------------------GGSVSSGNYYWTWIRQPPGKGLEWIGYIYYS--G
Sequ 5:    --------------------------GDSITSAGYYWTWIRQRPGKGLEWIGYIYYS--G
Sequ 6:    --------------------------GGSIKSGSHYWSWIRQSAGEGLEWIGRVYTT--G
Sequ 7:    --------------------------GFSFST--YWMTWVRLATGKGLEWVANIKED--G
Sequ 8:    --------------------------GDSISSGGYYWSWIRQHPGKGLEWIGYISYS--G
Sequ 9:    --------------------------GLTFSS--YGMHWVRQAPGKGLEWVALLSYD--G
Sequ 10:   --------------------------GGSISSGNYYWSWIRQHPGEGLEWIGCISNG--G

           60        70        80        90        100
           89012345678901234567890123456789012345678901      2     3456
           aaaaaaaaaaaaaa..a.a.aaaaa.aaa..aaa...a...aaa      a     aaaa
3m8o_HC:   DATAYAASMRGRLTISRDDSKNTAFLQMNSLKSDDTAMYYCVIR------G-----DVYN 106
1iga_HC:   GNTKYSQKFRGRVTFTRDTSATTAYMGLSSLRPEDTAVYYCARDP--YGGG-----KSEF 109
Sequ 1:    SGEYYLDSVKGRFAISRDNAKNSLYLQLNSLRAEDTAVYYCVRT--LRGDGG--------
Sequ 2:    SGEYYLDSVKGRFTISRDNAKNSLYLQLNSLRAEDTAVYYCVRT--LRGDGG--------
Sequ 3:    S-TYYNPSLKSRLTMSVDTSKDQFSLKLTSVTAADTAQYYCARA--LIQGLVREGYYYYM
Sequ 4:    S-TNYNPSLKSRVTISMDKSKNQFSLRLPSVTAADTAVYYCAR---YYDDLT--G-YFYF
Sequ 5:    S-TYYTPSLKSRVTISVDTSKNQFFLNLSSVTAADTAVYYCARN--FWSGPG------TR
Sequ 6:    G-TNKNPSLKSRVTISVDTSKNQFSLEPTSVTAADTAVYYCARD--LASGSG-----WGF
Sequ 7:    RDKYYVDSVRGRFTISRDNAKNSVYLQMNSLRPEDTAVYYCARR--VRPD----------
Sequ 8:    N-TYYNPSLERPVNISMDTSKNQLSLKLTSVTAADTAIYYCARSP-PGYSTG----YYRF
Sequ 9:    SNRYYADSVKGRFTISRDNSKNTLYLQMNSLRAADTAVYYCVRG--PRYCGGGSCSQYGM
Sequ 10:   S-TYYNPSLNDRLTISVDSANNQFSLKLSSVTAADTAVYYCARESLLYYGSH-LHHYYGM

            110       120
           78901234567890
           aa......
3m8o_HC:   RQWGQGTLVTVSSASPTSPKVFPLSLCSTQPDGNVVIACLVQGFFPQEPLSVTWSESGQG 166
1iga_HC:   DYWGQGTLVTVSSASPTSPKVFPLSLCSTQPDGNVVIACLVQGFFPQEPLSVTWSESGQG 169
Sequ 1:    SYWGQGILVTVSSA----------------------------------------------
Sequ 2:    SYWGQGILVTVSSA----------------------------------------------
Sequ 3:    DVWGEGTTVTVSSA----------------------------------------------
Sequ 4:    GYWGQGTLVTVSSA----------------------------------------------
Sequ 5:    DVWGKGTTVTVSSA----------------------------------------------
Sequ 6:    DYWGQGILVTVSSA----------------------------------------------
Sequ 7:    -YWGQGTLVTVSSA----------------------------------------------
Sequ 8:    DPWGQGTLVTVSSA----------------------------------------------
Sequ 9:    DVWGQGTTVTVSSA----------------------------------------------
Sequ 10:   DVWGQGTTVTVSSA----------------------------------------------
```

b

```
                    10        20        30         40        50
           12345678901234567890123456789012 345678901234567890123456789
           aaa.a...a...aSa.aa.a.a.....aaaaa aaaaa.a..aa...aa.aa...aa.aa
3m8o_LC:   DIVMTQSPLSLSVTPGEPASISCRSSQSLLRR-DGHNDLEWYLQKPGQSPQPLIYLGSTR 59
1iga_LC:   ELVMTQSPSSLSASVGDRVNIACRASQGIS------SALAWYQQKPGKAPRLLIYDASNL 54
Sequ 1:    -IVMTQSPSTLSASVGDRVTITCRASQSIS------SCLAWYQQKPGKAPKLLVYEASTL
Sequ 2:    -FQLTQSPATLSLSPGERATLSCRASQSVS------SYLAWFQRKPGQAPRLLIYDVSNR
Sequ 3:    DARLTQSPSSLSASLGDRVTITCRASQSII------NYLNWYQEKPGKAPKLLIYGASIL
Sequ 4:    -IVLTQSPATLSLSPGERATLSCRASQSIN------IYLAWYQQKPGQAPRLLIYEASNR
Sequ 5:    DIVMTQSPDSLAVSLGGRATINCKASQSVLYDGNNENYLAWYQQKPGQPPKLLIYWASTR
Sequ 6:    FIQLTQSPDSLAVSLGERATINCKSSQSILYTSNNKNYLAWYQQKPGQPPKLLIYLASTR
Sequ 7:    DIVMTQSPDSLAVSLGERATINCKSSQSVLYNSNNKNYLAWFQQKPGQPPNLVIYWASTR
Sequ 8:    DIVMTQSPDSLAVSLGERAPINCKSSQSVLYSSNNKNYLAWYQQKPGQPPKLLIYWASTR
Sequ 9:    DIQMNQSPSTLSASVGDRVTITCRASQSFS------EWLAWYQQKPGKAPKLLIYRASTL
Sequ 10:   DIQMTQSPSSLSASVGDRVTITCRASQSIS------SYLNWYQHKPGRAPKLLIHAASTL

           60        70        80        90        100         110
            0123456789012345678901234567890123456789 0. 1234567890123456789
            ..a..a......a....aaa.aa......aa...a.aaaaa  a...a...a.a.
3m8o_LC:    ASGVPDRFSGSGSGTDFTLKIIRVEAEDAGTYYCMQNKQTP--LTFGQGTRLEIKRTVAA 117
1iga_LC:    ESGVPSRFSGSGSGTDFTLTISSLQPEDFAIYYCQQFNSYP--LTFGGGTKVEIKRTVAA 112
Sequ 1:     ESGVPSRFSGSGSGTEFTLTISSLQPDDFATYYCQQYNSCS--RTFGQGTKVEIK-----
Sequ 2:     ATGIPARFSGSGPGTDFTLTISSLEPEDFAVYYCQQRSNWPPKLTFGGGTKVEIK-----
Sequ 3:     QSGVPSRFSATGYGTDFTLTISSLQPEDLANYFCQQSYKTP--YTFGQGTKVEIK-----
Sequ 4:     ATGIPARFSGSGSGTDFIRTISSLEPEDFAVYYCQHRRSWP--LTFGGGTKVDIK-----
Sequ 5:     ESGVPDRFSGSGSGTDFTLTISDLQAEDVAVYYCQQCFSTP--WTFGQGTKVEYQT----
Sequ 6:     ESGVPDRFSGSGSGTDFTLTINSLQAEDVAVYYCQQYYIYP--ITFGGGTKVDIK-----
Sequ 7:     ESGVPDRFSGSGSGTDFTLTINSLQAEDVAVYFCLQYYSTP--LTFGQGTQVEIK-----
Sequ 8:     ESGVPDRFSGSGSGTDFTLTISSLQAEDVAVYYCQQYYSTPP-YSIGQGTKVDIK-----
Sequ 9:     KSGVPSRFSGSGSGAEFTLTISGLQPDDFATYYCQQYRSFP--WTFGQGTKGDIK-----
Sequ 10:    HSEVPSRFSGSGSGTDFTLTISSLQPEDSATYYCQRTYHTP--LTFGGGTKVDIKR----
```

c

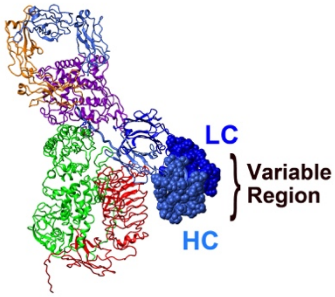

Supplementary Figure 3

Supplementary Fig. 3. **Identifying variable residues within the bound IgA1 FAB**. Sequence alignments are shown for the IgA1 variable regions, which include a) the heavy chain (HC) and b) the light chain (LC). Variable residues within the FaB (delineated as "a") were identified by this sequence alignment and the corresponding residues in the IgA1P-E1605A/IgA1 complex were changed to alanine. Sequence alignments included the initial FAB fit (PDB accession 3M8O), the full mode of IgA1 (PDB accession 1IGA), and 10 additional sequences from the variable region obtained from the NCBI database. c) Variable residues mutated to alanine shown as balls for both LC (dark blue) and HC (light blue) mapped onto the IgA1P-E1605A/IgA1 complex.

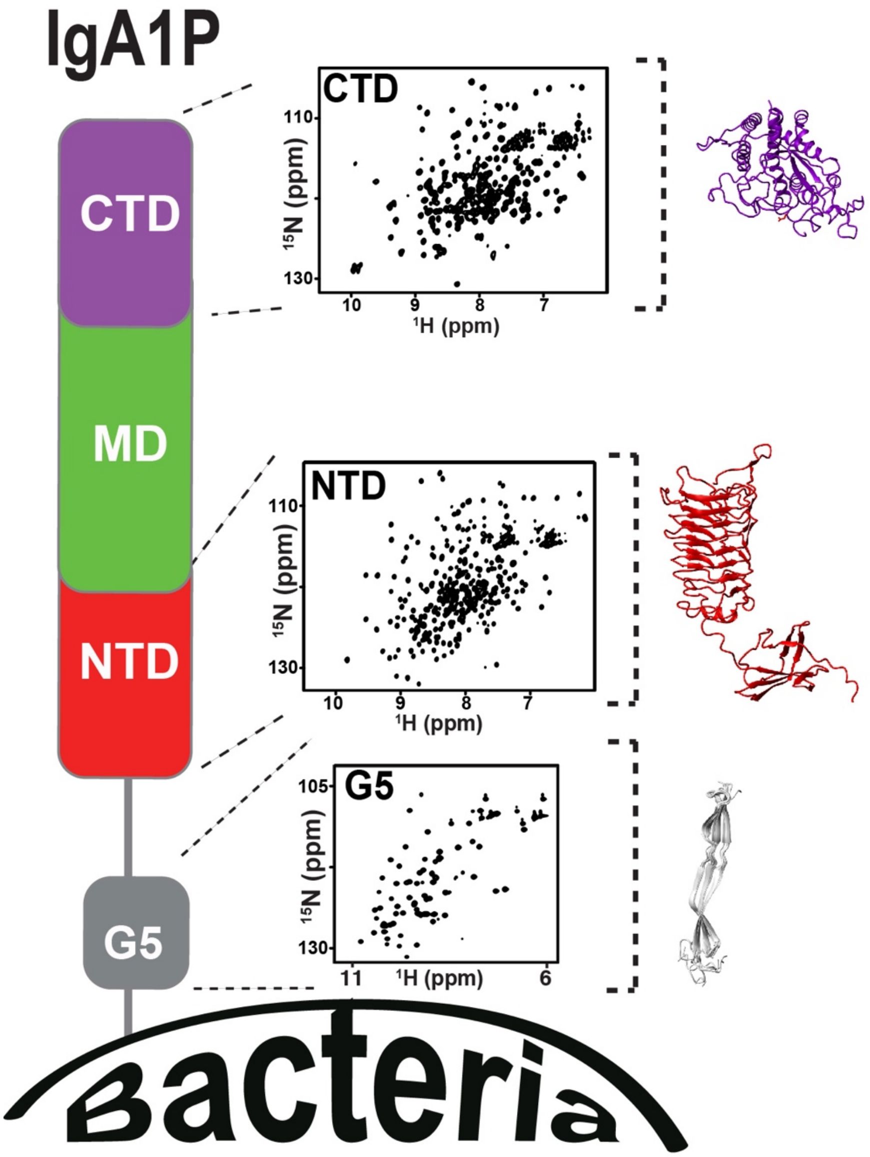

Supplementary Figure 4

Supplementary Fig. 4. **Domains of IgA1P are independently folded**. The mature IgA1P structure is depicted (residues 154-1963) with HSQC spectra of individually purified domains that include the G5, the NTD from the protease region, and the CTD from the protease region.

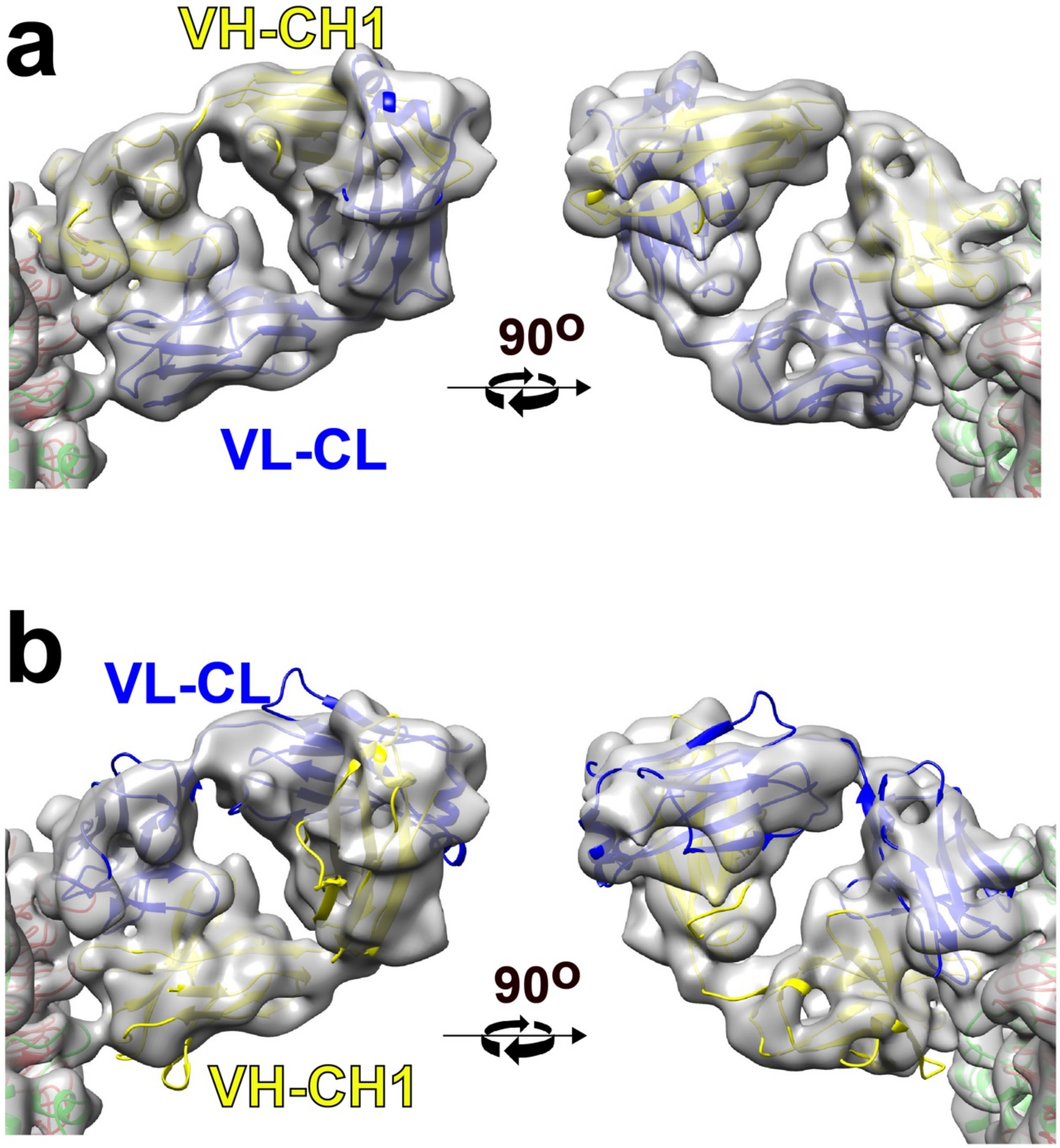

Supplementary Figure 5

Supplementary Fig. 5. **Rigid body fitting of the mAb to the 3D density map of IgA1P/mAb**. The density of the IgA1P/mAb complex is shown in Chimera at a contour level of 0.136. a) The initial model of the Fab fit with the LC (blue) on the bottom and HC (yellow) on top, which results in 337/3230 atoms unable to fit within the density. b) The identical view but with the LC and the HC subunits switched, which results in 1385/3230 atoms unable to fit within the density.

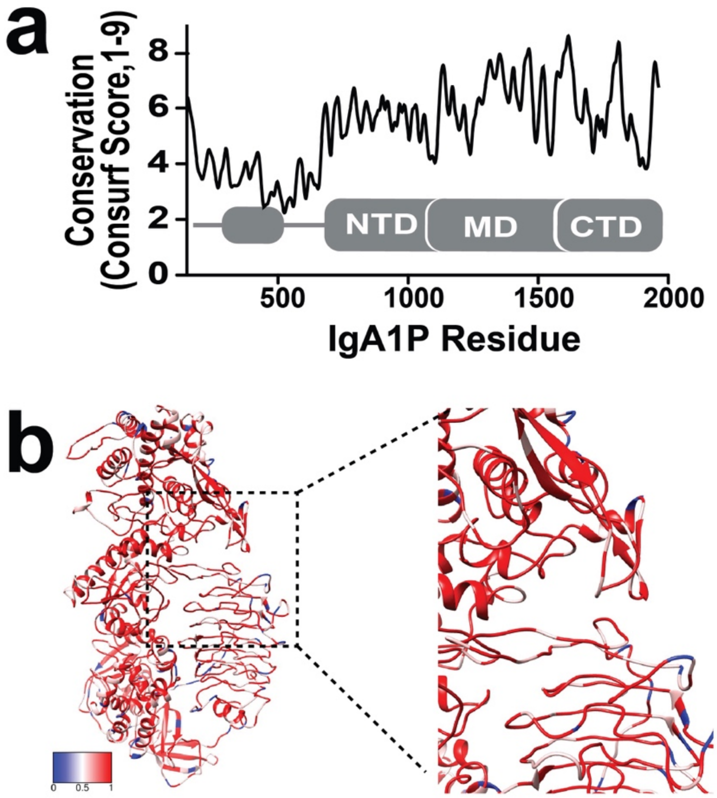

Supplementary Figure 6

Supplementary Fig. 6. **Sequence similarity of *S. pneumoniae* IgA1P with other bacterial metalloproteases.** a) Conservation of IgA1P across over 140 proteins that comprise multiple bacterial strains as reported by ConSurf {Ashkenazy, 2016 #1996}. ConSurf scoring is reported as 0-9 (low-high conservation) and the domains of mature IgA1P residues 154-1963 are shown. It should be noted that this search reveals other similar metalloproteases that likely comprise similar structural folds but cleave different host targets, such as ZmpB and ZmpC {Bek-Thomsen, 2012 #2552}. b) Sequence identity of *S. pneumoniae* IgA1P is compared to *Streptococcus oralis* (uniprot accession P96470, 88.2%) and *Streptococcus sanguinis* (uniprot accession Q59976, 74.7%), where a value of 0 (blue) indicates all three sequences are different , 0.5 (white) indicates one is identical and one is different, and 1 (red) indicates complete conservation. Residues were colored in Chimera and the active site recognized by the neutralizing mAb (mAb #2) is further shown as a blow up (dotted rectangle).

	IgA1P	IgA1P+IgA1	IgA1P+mAb
**Data Collection and Processing**			
Microscope	Arctica	Titan Krios	Titan Krios
Voltage (kV)	200	300	300
Magnification (nominal)	45,000	81,000	81,000
Electron Dose ($e^-/Å^2$)	~62	47	57
Camera	Gatan K3	Falcon 3	Falcon 3
Defocus range (um)	-1~ -2.2	-0.9 ~ -2.4	
Pixel size (Å)	0.864	1.059	0.832
Movies collected	4612	2467	2516
Symmetry imposed	C1	C1	C1
Final particle images (no.)	293,002	93,218	64,968
Map resolution (Å)	3.77	3.49	4.83
Sharpening B-factor ($Å^2$)	-165.2	-81.6	-226.3
Software used to process data	RELION	cryoSPARC	cryoSPARC
**Refinement statistics**			
Number of protein atoms (non-H)	10071	16218	13325
R.m.s. deviations			
Bonds (Å)	0.007	0.008	0.013
Bond angles (º)	1.020	1.006	2.214
Validation			
MolProbity score	2.09	2.92	2.70
Clash score	9.63	14.77	6.49
Poor rotamers (%)	1.02	1.26	2.68
Ramachandran plot			
Favored (%)	91.59	89.95	91.64
Allowed (%)	8.41	9.58	7.12
Disallowed (%)	0	0.47	1.24
C-beta deviations	0	0	0
Model vs Data CC	0.83	0.70	0.88
FSC model (0.143)	3.7	3.8	4.8
EMDB access code	22205	22204	22328
PDB access code	6XJB	6XJA	7JGJ

Supplementary Table 1: Cryo-EM data collection, refinement and validation statistics.

Supplementary References

1 Chi, Y. C. *et al.* Streptococcus penumoniae IgA1 protease: A metalloprotease that can catalyze in a split manner in vitro. *Protein Science* **26**, 600-610 (2017).

2 Paukovich, N. *et al.* Streptococcus pneumoniae G5 domains bind different ligands. *Protein Science* **28**, 1797-1805 (2019).

3 Scheres, S. H. W. in Resolution Revolution: Recent Advances in Cryoem Vol. 579 Methods in Enzymology (ed R. A. Crowther) 125-157 (2016).

4 Schulz, E. C. & Ficner, R. Knitting and snipping: chaperones in beta-helix folding. *Current Opinion in Structural Biology* **21**, 232-239 (2011).

5 Emsley, P., Lohkamp, B., Scott, W. G. & Cowtan, K. Features and development of Coot. *Acta Crystallographica Section D-Biological Crystallography* **66**, 486-501 (2010).

6 Zheng, S. Q. *et al.* MotionCor2: anisotropic correction of beam-induced motion for improved cryo-electron microscopy. *Nature Methods* **14**, 331-332 (2017).

7 Zhang, K. Gctf: Real-time CTF determination and correction. *Journal of Structural Biology* **193**, 1-12 (2016).

8 Adams, P. D. *et al.* PHENIX: a comprehensive Python-based system for macromolecular structure solution. *Acta Crystallographica Section D-Biological Crystallography* **66**, 213-221 (2010).
