# Supplementary material for: Mechanism and inhibition of Streptococcus pneumoniae IgA1 protease
Source: Nat Commun. 2020 Nov 27;11:6063. doi: 10.1038/s41467-020-19887-3 (PMC7695701; doi:10.1038/s41467-020-19887-3)
Supplement: Supplementary file 3 — Reporting Summary [file 41467_2020_19887_MOESM3_ESM.pdf]

nature research

Corresponding author(s): Eisenmesser, Elan

Last updated by author(s): Oct 30, 2020

Reporting Summary

Nature Research wishes to improve the reproducibility of the work that we publish. This form provides structure for consistency and transparency in reporting. For further information on Nature Research policies, see our Editorial Policies and the Editorial Policy Checklist.

Statistics

For all statistical analyses, confirm that the following items are present in the figure legend, table legend, main text, or Methods section.

n/a	Confirmed	
☐	☒	The exact sample size (n) for each experimental group/condition, given as a discrete number and unit of measurement
☒	☐	A statement on whether measurements were taken from distinct samples or whether the same sample was measured repeatedly
☒	☐	The statistical test(s) used AND whether they are one- or two-sided<br>*Only common tests should be described solely by name; describe more complex techniques in the Methods section.*
☒	☐	A description of all covariates tested
☒	☐	A description of any assumptions or corrections, such as tests of normality and adjustment for multiple comparisons
☐	☒	A full description of the statistical parameters including central tendency (e.g. means) or other basic estimates (e.g. regression coefficient) AND variation (e.g. standard deviation) or associated estimates of uncertainty (e.g. confidence intervals)
☒	☐	For null hypothesis testing, the test statistic (e.g. F, t, r) with confidence intervals, effect sizes, degrees of freedom and P value noted<br>*Give P values as exact values whenever suitable.*
☒	☐	For Bayesian analysis, information on the choice of priors and Markov chain Monte Carlo settings
☒	☐	For hierarchical and complex designs, identification of the appropriate level for tests and full reporting of outcomes
☒	☐	Estimates of effect sizes (e.g. Cohen's d, Pearson's r), indicating how they were calculated

Our web collection on statistics for biologists contains articles on many of the points above.

Software and code

Policy information about availability of computer code

Data collection	Leginon 3.3
Data analysis	Gctf:1.06, MotionCor2:1.1, CTFFind4, Rel ion 3.0, CryoSPARC v2.10, Bsoft 1.9.0, gCTF, Phenix 1.10.1, Coot 0.8.9.1, Chimera 1.14.

For manuscripts utilizing custom algorithms or software that are central to the research but not yet described in published literature, software must be made available to editors and reviewers. We strongly encourage code deposition in a community repository (e.g. GitHub). See the Nature Research guidelines for submitting code & software for further information.

Data

Policy information about availability of data

All manuscripts must include a data availability statement. This statement should provide the following information, where applicable:

- Accession codes, unique identifiers, or web links for publicly available datasets
- A list of figures that have associated raw data
- A description of any restrictions on data availability

The cryo-EM maps are deposited in the Electron Microscopy Data Bank under accession codes EMD-22205 (IgA1P residues 665-1963), EMD-22204 (IgA1P residues 665-1963 with the single E1605A mutation in complex with IgA1), EMD-22328 (IgA1P residues 665-1963 in complex with the mAb). Structure coordinates are deposited at the Protein Data Bank with accession codes 6XJB (IgA1P residues 665-1963), 6XJA (IgA1P residues 665-1963 with the single E1605A mutation in complex with IgA1), 7JGJ (IgA1P residues 665-1963 in complex with the mAb). Source data is provided with this paper.

Field-specific reporting

Please select the one below that is the best fit for your research. If you are not sure, read the appropriate sections before making your selection.

☒ Life sciences ☐ Behavioural & social sciences ☐ Ecological, evolutionary & environmental sciences

For a reference copy of the document with all sections, see nature.com/documents/nr-reporting-summary-flat.pdf

Life sciences study design

All studies must disclose on these points even when the disclosure is negative.

Sample size	This is a single particle reconstruction
Data exclusions	No data was excluded from analysis
Replication	Replication for neutralization and mAb binding was used (3x or more), but replication is NOT applicable to single particle reconstruction that comprises multiple particles.
Randomization	There is nothing to randomize.
Blinding	Neutralization and binding for Figure 4 were collected quadruplicate and triplicate, respectively, as provided in Source Data.

Reporting for specific materials, systems and methods

We require information from authors about some types of materials, experimental systems and methods used in many studies. Here, indicate whether each material, system or method listed is relevant to your study. If you are not sure if a list item applies to your research, read the appropriate section before selecting a response.

Materials & experimental systems

n/a	Involved in the study	
☐	☒	Antibodies
☒	☐	Eukaryotic cell lines
☒	☐	Palaeontology and archaeology
☐	☒	Animals and other organisms
☒	☐	Human research participants
☒	☐	Clinical data
☒	☐	Dual use research of concern

Methods

n/a	Involved in the study	
☒	☐	ChIP-seq
☒	☐	Flow cytometry
☒	☐	MRI-based neuroimaging

Antibodies

Antibodies used	Monoclonal antibody purified from a single hybridoma produced using Streptoccocus Pneumoniase IgAl Protease residues 154-1963 through the University of Colorado Cancer Center. , Anti-IgA1 CH3, Alkaline phosphatase labeled goat anti-human Kappa
Validation	Monoclonal antibody was validated by both binding and neutralization of Streptoccocus Pneumoniase IgAl Protease activity. As described in Figure 4, the mAb both neutralizes IgA1 protease activity (Figure 4a) and competes with binding to the IgA1 substrate (Figure 4b).

Animals and other organisms

Policy information about studies involving animals; ARRIVE guidelines recommended for reporting animal research

Laboratory animals	7-9 week old, Balb C, female mice.
Wild animals	No wild samples were used in this study.
Field-collected samples	No Field collected samples were used in this study.
Ethics oversight	The "Standardized Protocol for Production of Monoclonal Antibodies in Mice", protocol # 104513(12)1C, was approved by the Animal Care and Use Committee, accredited by the Association for Assessment and Accreditation of Laboratory Animal Care – File Number 00235. We have complied with all relevant ethical regulations for animal testing and research.

Note that full information on the approval of the study protocol must also be provided in the manuscript.
